# Heterogeneous response of estimated insulin sensitivity indices to metformin in young individuals with type 1 diabetes and different phenotypes

**DOI:** 10.1186/s13098-024-01451-4

**Published:** 2024-09-02

**Authors:** Luana A. L. Ramaldes, Sarah S. dos Santos, Patricia M. Dualib, Joao R. de Sa, Sérgio A. Dib

**Affiliations:** 1 Department of Medicine, Division of Endocrinology, Diabetes Center, Escola Paulista de Medicina, Universidade Federal de São Paulo, São Paulo, SP Caixa Postal 20266 CEP 04022-001 Brazil; 2 Division of Medicine-Endocrinology, ABC School of Medicine, São Paulo, Brazil

**Keywords:** Insulin resistance, Type 1 diabetes mellitus, Obesity, Metformin

## Abstract

**Aims:**

This study aimed to investigate whether the response to adding metformin to insulin in young adults with type 1 diabetes (T1D) differs according to weight phenotype and insulin sensitivity index.

**Methods:**

A prospective pilot study was conducted over 26 weeks in which insulin plus metformin (2 g/day) was administered to 35 individuals, ranging from normal weight (NW) to overweight (OW) to obese (OB) T1D individuals, to correlate insulin sensitivity indices and other clinical variables.

**Results:**

At the end of the follow-up period, all groups showed an increase in the eGDR (NW: 7.37 vs 8.16, p = 0.002; OW: 7.28 vs 8.24, p < 0.001; OB: 6.33 vs 7.52 p < 0.001). K_ITT_ and SEARCH SCORE improved only in the OB group (2.15 vs 3.14, p < 0.001 and 5.26 vs 5.72, p = 0.007, respectively). Furthermore, HbA1c and BMI were significantly greater in the OB group (− 0.62%, p < 0.001; − 1.12 kg/m^2^, p = 0.031, respectively). Regression analysis revealed that the serum levels of triglycerides and uric acid were significantly (0.059, p = 0.013; 0.076, p = 0.001) associated with insulin sensitivity indices.

**Conclusions:**

The study showed that eGDR improved independently of basal weight after metformin treatment. However, the K_ITT_ and SEARCH indices improved only in the obese group. Triglycerides and uric acid are associated with insulin sensitivity indices. These results highlight the heterogeneity of the mechanisms underlying insulin resistance and its response to metformin in individuals with T1D.

## Introduction

Type 1 diabetes mellitus (T1D) is a chronic autoimmune disease characterized by the destruction of beta cells, leading to insulin deficency [1]. Traditionally, individuals with T1D were characterized by low or normal weight. However, in recent years, T1D has become a more heterogeneous disease, and many studies have reported an increase in the prevalence of overweight and obese individuals in this group [2, 3]. This change was made evident during a study by the Diabetes Control and Complications Trial/Epidemiology of Diabetes Interventions and Complications (DCCT/EDIC) Research Group, which showed an increase in the prevalence of overweight from 2% at baseline to 33–36% after 18 years of follow-up [4]. Similarly, our Brazilian multicenter study of T1D reportes a prevalence of 20.4% for overweight and 9.8% for obesity in patients within five years of diagnosis [5]. Other studies evaluating overweight/obese patients with T1D have shown an increase in the prevalence of microvascular and macrovascular complications [6], highlighting the importance of addressing other metabolic factors behind glycemic control in these individuals. In this context, insulin resistance in T1D, as in type 2 diabetes (T2DM), has been increasingly recognized to play an important role in the pathophysiology of cardiovascular disease (CVD) [7], but the underlying mechanisms are not fully understood, as in T2DM [6, 8].

Metformin is a safe drug that reduces insulin resistance (IR) and BMI in young people with prediabetes [8] and T2DM and reduces the risk of CVD and mortality in adults [9]. Although guidelines from the United States and the United Kingdom have suggested adding metformin to insulin therapy in T1D [10, 11], its use in routine clinical practice for T1D is not widespread. However, the use of metformin in treating T1D has shown controversial results [12, 13]. This is largely due to the variability in response to metformin, which depends significantly on the patient's phenotype, particularly their weight status. This heterogeneity underscores the need for a personalized approach to treatment, which may not be feasible in all clinical settings due to the requirement for specialized knowledge and resources. Moreover, the lack of large-scale, long-term randomized controlled trials confirming the benefits of metformin in T1D has limited its acceptance and incorporation into standard care protocols. Therefore, our study aimed to characterize a specific phenotype of T1D where the use of metformin might be more appropriate, focusing on its efficacy as an adjunctive therapy in different weight categories.

We conducted a 26-week short-term randomized pilot study to assess whether the response of young adults with T1D to the addition of metformin to insulin differs according to their weight phenotype and insulin sensitivity index. This study is particularly relevant in the context of the rising prevalence of overweight and obesity in T1DM, as it aims to determine if metformin can effectively address the associated insulin resistance and improve overall metabolic control.

## Methods

We conducted prospective study that followed 35 participants with T1D in an outpatient clinic of the Diabetes Center of the Federal University of São Paulo, SP, Brazil.

The inclusion criteria included people with T1D aged 18 to 35 years, HbA1c ≤ 9.0% (75 mmol/mol), and who had lived with the disease for at least 10 years. T1D was defined as a diagnosis of diabetes before age 30 and insulin use within 1 year after diagnosis. Forty-three participants with T1D were recruited, and 35 (81.3%) completed the study. Participants were divided into three groups according to BMI: normal weight (BMI < 25 kg/m^2^, n = 10), overweight (BMI 25–29.9 kg/m^2^, n = 12), and obese (> 30 kg/m^2^, n = 13). The exclusion criteria included HbA1c > 9% (75 mmol/mol), treatment with other drugs (excluding insulin), creatinine clearance less than 60 mL/min/173 m^2^, liver failure, breastfeeding, pregnancy, and a history of severe hypoglycemia.The decision to exclude individuals with HbA1c > 9% was made to reduce variability related to poor glycemic control, which could confound the assessment of insulin sensitivity.

The Institutional Ethics Committee of the Federal University of São Paulo approved the study (CAAE NUMBER: 84,245,718,500,005,505), and informed consent was obtained from the participants.

All participants received subcutaneous basal/bolus insulin through a multiple daily insulin (MDI) regimen. After a run-in period of at least 4 weeks, extended-release metformin (2 g/day) was added to the insulin treatment group. The daily dose of metformin in the study continued to increase for 4 weeks, with an initial dose of 500 mg until the goal of 2000 mg, which remained for 26 weeks. Those who had already used the medication discontinued it three weeks before the initial analysis and examination collection. This washout period is equivalent to approximately 15 terminal half-lives for the elimination of metformin from the erythrocyte and plasma compartments and represents multiple doses. The insulin dose was adjusted based on clinical judgment, and capillary blood glucose measurements were collected at least 4 points a day.

Patients were evaluated between 8:00 and 9:00 am after fasting overnight. Anthropometric measurements, body composition data (bioelectrical impedance analysis—InBody270®, Biospace, California, USA) and total daily insulin dose data were collected at baseline and at 13 and 26 weeks. Biochemical tests (glycated hemoglobin (HPLC), serum lipids (total cholesterol, high-density lipoprotein cholesterol [HDL-c], low-density lipoprotein cholesterol [LDL-c], triglycerides: colorimetric peroxidase assay), uric acid (fluorescent uricase assay), ferritin (chemiluminescence), ultrasensitive C-reactive protein (CRP) (turbidimetric assay), and glucose (colorimetric enzyme assay)) and insulin sensitivity indices were performed at baseline and 26 weeks after the start of metformin treatment.

The insulin tolerance test was performed using the method described by Bonora et al. [14]. The test involved administering a bolus injection of regular human insulin at 0.1 U/kg and collecting blood samples to measure glucose levels and calculate the decay rate 3 to 15 min after insulin infusion (K_ITT_) using the formula 0.693/t1/2 and the least squares method. This test was performed at baseline and 26 weeks after metformin treatment.

Furthermore, two other tests were used to estimate insulin sensitivity: a) the estimated glucose disposal rate (eGDR: 24.31–(12.22 × waist-to-hip ratio [WHR])–(3.29 × hypertension [0 = no, 1 = yes])–(0.57 × HbA1c [%])) [15] and b) the SEARCH IS score: eIS exp (4.64725–0.02032 [waist, cm]− 0.09779 [HbA1c, %]− 0.00235 [triglycerides, mg/dL]) [16]. These last two tests were evaluated at baseline and 26 weeks after metformin treatment.

The statistical analysis used a confidence level of 95% and a statistical power of 80% (GPower v.3.1.9.2, Universität Kiel, Germany). Two-way nonparametric ANOVA and one-way nonparametric ANOVA were used for comparisons between and within groups, respectively. Mixed linear model analysis was used to assess the associations between variables at baseline and after 26 weeks of treatment. The level of significance adopted in the tests was 0.05. We used R software version 3.6.0 to perform all analyses.

## Results

Thirty-five patients with T1D were studied. The mean age (SD) of the participants was 25.2 (4.4) years. Eighteen of them (51%) were women, and 57% were white. HbA1c was 8.19 (0.54) % or 66 mmol/mol (6.07), BMI was 27.4 (4.15) kg/m^2^, and total daily insulin was 0.81 (0.2) U/kg. These baseline characteristics were similar across the groups, ensuring that any observed differences in outcomes could be attributed primarily to the intervention.

Individuals with T1D were divided according to their body weight into three groups: normal weight (n = 10), overweight (n = 12) and obese (n = 13). The duration of T1D was 13 (4.1) years in the normal weight group, 17 (5.4) years in the overweight group, and 15 (3.6) years in the obese group. (Table 1).

**Table 1:** Baseline Clinical and Metabolic Characteristics of Patients with Type 1 Diabetes

	Normal weight (NW) (n = 10)	Overweight (OW) (n = 12)	Obese (OB) (n = 13)	*p value*^b^	NW vs. OW *p value*^c^	NW vs. OB *p value*^c^
Female sex, N (%)^a^	4 (40%)	5 (42%)	9 (69%)	0.280	–	–
Age, years	23 (4.5)	26 (4.4)	25 (4.4)	0.503	–	–
Duration of diabetes, years	13 (4.1)	17 (5.4)	15 (3.6)	0.215	–	–
Ethnic origin- white	9 (90)	5 (42)	6(46)	0.06	–	–
Other	1(10)	7(58)	7(54)			
Family history of Type 2 diabetes, N (%)	7 (70)	7 (58)	8 (61)	0.909	–	–
Family history of Type 1 diabetes, N (%)	1 (10)	5 (41)	0	**0.011**	0.486	1.305
BMI, kg/m^2^	21.8 (1.8)	27.8 (1.2)	31.3 (1.5)	** < 0.001**	** < 0.001**	** < 0.001**
Waist circumference (cm)	78.4 (3.8)	90.5 (5.8)	96.6 (9.0)	** < 0.001**	** < 0.001**	** < 0.001**
Visceral adipose tissue (cm^2^)	46.1 (24)	88.8 (28.3)	96.1 (30.2)	**0.001**	**0.006**	** < 0.001**
HbA1c % of total hemoglobin (mmol/mol)	8.0 (0.7) 64	8.1 (0.4) 65	8.3 (0.4) 67	0.369	–	–
total daily insulin, U/kg per day	0.82 (0.15)	0.93 (0.27)	0.71 (0.14)	0.051	–	–
Systolic blood pressure (mmHg)	121.9 (9.8)	120.7 (7.9)	132.3 (13.4)	**0.021**	0.966	**0.05**
Diastolic blood pressure (mmHg)	74.1 (7.1)	76.2 (11.6)	83 (5.4)	**0.016**	1	**0.003**
Total cholesterol (mg/dL)	173.2 (36.3)	190.6 (28.3)	193.6 (40.6)	0.365	–	–
LDL cholesterol (mg/dL)	103.9 (25.2)	112.7 (24.6)	118.5 (34.1)	0.487	–	–
HDL cholesterol (mg/dL)	48.9 (15.1)	61 (19.4)	55.8 (10.8)	0.173	–	–
Triglycerides (mg/dL)	70.7 (19.9)	93.3 (35.9)	95.4 (46.6)	0.299	–	–
Antihypertensive drugs, N (%)	0	2 (17)	3 (23)	0.399	–	–
Statin, N (%)	1 (10)	4 (33)	3 (23)	0.465	–	–

^a^Data are presented as the mean (SD: standard deviation) or N (%)

^b^Kruskal-Wallis test and Fisher's exact test Tukey's test and Fisher's

^c^Exact Test with Bonferroni correction

**Fig. 1:**
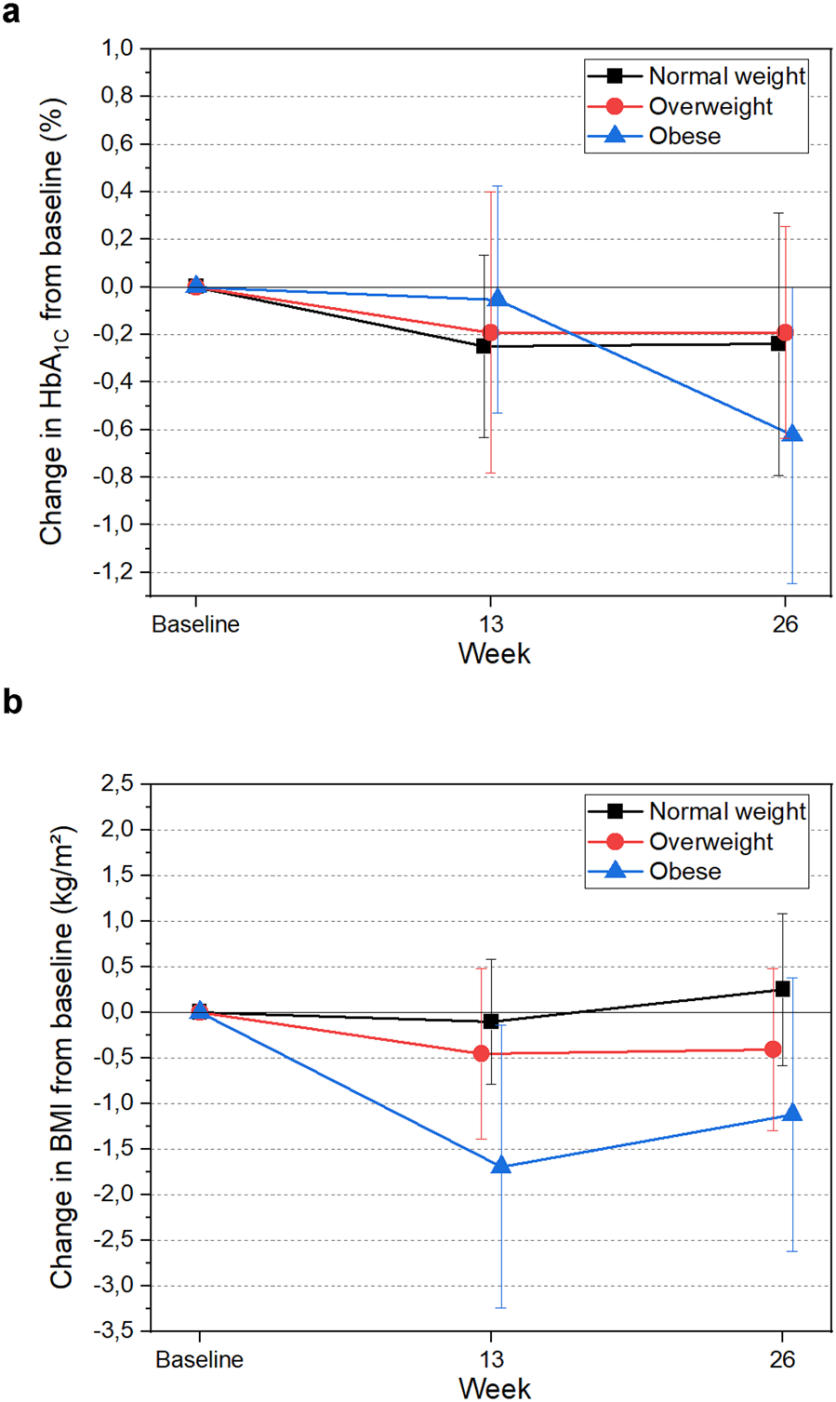
Changes from baseline in HbA1c and BMI after metformin use in the T1D group with different BMIs. Mean change from baseline to 26 weeks for (**a**) HbA_1c_ (OB-p = 0.004) and (**b**) BMI (OB-p = 0.031)

The baseline HbA1c was similar in the three groups of patients with T1D studied. However, after 26 weeks of metformin treatment, we found a significant reduction in HbA1c in the obese group (− 0.62%, p < 0.001) (Table 2/Fig. 1A). These changes occurred despite the lack of a significant reduction in insulin dose per kg of body weight, suggesting that metformin's benefits in this population might be related to enhanced insulin sensitivity and metabolic control rather than a simple reduction in insulin requirements.

**Table 2:** Comparison of changes in clinical and laboratory results

	Normal weight (n = 10)			Overweight (n = 12)			Obese (n = 13)			NW vs. OW	NW vs. OB
	Baseline	26 weeks	p value^a^	Baseline	26 weeks	p value^a^	Baseline	26 weeks	p value^a^	Basal At 26 weeks p value^b^	Basal At 26 weeks p value^b^
BMI (kg/m^2^), SD	21.83 (1.86)	22.08 (1.74)	0.374	27.82 (1.22)	27.41 (1.51)	0.139	31.31 (1.52)	30.19 (2.27)	**0.031**	**0.007**	**0.006**
Body weight (kg),	62.78 (9.62)	63.62 (8.62)	0.463	78.60 (8.18)	77.23 (7.59)	0.101	85.58(9.01)	82.57 (10.26)	**0.021**	0.302	**0.016**
Waist-hip ratio	0.83 (0.05)	0.83 (0.03)	0.558	0.87 (0.06)	0.87 (0.05)	0.940	0.90 (0.07)	0.90 (0.07)	0.191	0.593	0.945
percent body fat (%),	22.50 (7.93)	23.62 (9.28)	0.228	32.81 (7.34)	32.05 (7.48)	0.371	37.71 (7.12)	37.26 (7.39)	0.576	0.133	0.135
Visceral adipose tissue, cm^2^	46.17 (24.04)	50.96 (19.19)	0.221	88.84 (28.35)	86.86 (26.66)	0.144	96.13 (30.20)	92.52 (31.19)	0.310	**0.033**	0.068
Systolic blood pressure (mmHg)	121.9 (9.84)	118.5 (6.26)	0.424	120.7 (7.96)	117.2 (11.49)	0.413	132.3 (13.43)	127.8 (11.41)	0.088	0.965	0.966
HbA1c (%), mmol/mol	8.06 (0.70) 65	7.82 (0.73) 62	0.203	8.12 (0.46) 65	7.93 (0.53) 63	0.165	8.36 (0.49) 68	7.74 (0.69) 61	**0.004**	0.962	0.155
Total daily insulin per kg,	0.82 (0.15)	0.82 (0.16)	0.953	0.93 (0.27)	0.86 (0.25)	0.069	0.71 (0.14)	0.76 (0.16)	0.110	0.363	0.255
K_itt_, (%/min)	3.47 (1.20)	4.09 (0.73)	0.080	2.45 (1.11)	3.41 (1.41)	0.081	2.15 (1.18)	3.14 (1.34)	** < 0.001**	0.924	0.802
eGDR (mg/kg min)	7.37 (1.67)	8.16 (1.55)	**0.002**	7.28 (1.16)	8.24 (1.03)	** < 0.001**	6.33 (1.45)	7.52 (1.44)	** < 0.001**	0.090	**0.020**
SEARCH ISscore	7.54 (1.40)	7.89 (0.73)	0.400	6.08 (0.91)	6.30 (0.83)	0.495	5.29 (1.18)	5.72 (1.29)	**0.007**	0.804	0.985
Total Cholesterol (mg/dL)	173.20 (36.32)	173.60 (44.07)	0.946	190.67 (28.36)	199.08 (47.58)	0.461	193.62 (40.64)	191.62 (31.16)	0.814	0.822	0.980
LDL (mg/dL)	103.90 (25.20)	105.80 (31.37)	0.710	112.75 (24.68)	123.58 (37.10)	0.195	118.54 (34.16)	117.92 (28.42)	0.935	0.623	0.769
HDL (mg/dL)	48.90 (15.14)	50.80 (13.26)	0.330	61.00 (19.42)	57.17 (17.19)	0.476	55.85 (10.85)	54.62 (10.02)	0.684	0.124	0.314
Triglycerides (mg/dL)	70.70 (19.98)	83.50 (21.70)	0.081	93.33 (35.95)	90.00 (28.22)	0.772	95.46 (46.68)	97.15 (43.71)	0.756	0.219	0.215
PCR (mg/dL)	0.71 (1.13)	0.30 (0.42)	0.213	0.29 (0.19)	0.27 (0.20)	0.625	0.56 (0.63)	0.55 (0.41)	0.735	0.694	0.220
Uric acid (mg/dL)	4,18 (1.08)	3.94 (0.84)	0.256	4.08 (0.66)	4.13 (0.66)	0.744	4.07 (0.92)	4.08 (0.74)	0.951	0.395	0.705
Ferritin (ng/mL)	100.89 (46.71)	83.85 (39.40)	0.084	96.69 (32.28)	84.53 (28.51)	0.185	95.97 (63.73)	90.55 (60.40)	0.456	0.977	0.902

The data are presented as the mean and standard deviation. The effects of metformin treatment were compared among the normal weight, overweight, and obese groups

^a^One-way ANOVA and paired test T

^b^Two-way ANOVA

From baseline to 26 weeks after metformin treatment, the obese group experienced greater weight loss with metformin than did the normal weight group (− 3.0 vs + 0.54 kg; p = 0.016). Additionally, there was a significant reduction in BMI (− 1.12 vs + 0.25 kg/m^2^; p = 0.006) (Table 2/Fig. 1B), further underscoring the impact of metformin on managing weight in this subgroup.

**Fig. 2:**
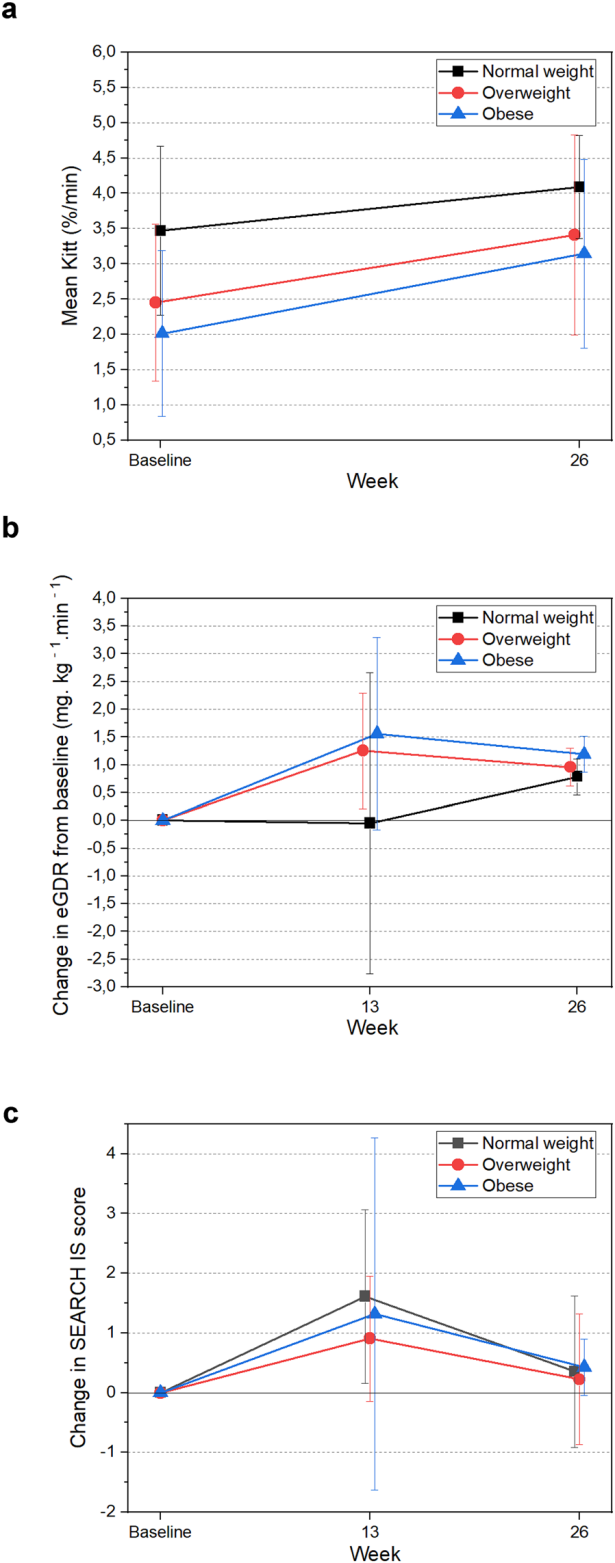
Changes from baseline in insulin sensitivity were evaluated using three different indices after metformin use in a group of T1D patients with different BMIs. **a** Mean from baseline at 26 weeks for the K_ITT_ (OB-p < 0.001)_._ **b** Mean change from baseline for 13 and 26 weeks for eGDR (NW-p = 0.002, OW-p < 0,001, and OB-p < 0.001) and (**c**) SEARCH IS score (OB-p = 0.007)

Insulin sensitivity, as evaluated by K_ITT_, improved across all T1D groups after 26 weeks of metformin treatment. However, the increase was more pronounced in the obese group (+ 0.99%/min, p < 0.001) than in the overweight group (+ 0.96%/min, p = 0.081) and the normal weight group (+ 0.62%/min, p = 0.08) (Fig. 2A). Similar patterns were observed when evaluating insulin sensitivity using other indices, such as the eGDR and the SEARCH IS. Compared with the overweight and normal weight groups, the obese group showed the greatest improvements in both the eGDR (+ 1.19 mg·kg− 1·min− 1, p < 0.001) (Fig. 2B) and the SEARCH IS score (+ 0.42, p = 0.007) (Table 2/Fig. 2C).

This study also explored the relationships between insulin sensitivity indices and other metabolic variables. Regression analysis, adjusted for age, sex, and baseline glycemic control, revealed that triglyceride and uric acid levels were significantly associated with insulin sensitivity indices, particularly in the obese group. A reduction of one unit in serum triglycerides was associated with a 0.01 increase in K_ITT_ (p = 0.008). Similarly, a variation of one unit in the serum uric acid level led to a change of − 0.45 (%/min) in the K_ITT_ (p = 0.014).

Cnages in the eGDR was also related to the levels of serum triglycerides and uric acid, in addition to the family history of systemic arterial hypertension and type 2 diabetes. The SEARCH-IS score was also shown to be related to serum uric acid in addition to serum ferritin, CRP, and visceral adipose tissue (Table 3).

**Table 3:** Variables associated with the change in insulin sensitivity indices (K_ITT_, eGDR, and SEARCH IS score) after 26 weeks of metformin

	K_ITT_		eGDR		SEARCH IS score	
	Coefficient^a^	*p* value	Coefficient^a^	*p* value	Coefficient^a^	*p* value
Triglycerides	− 0.01	**0.008**	− 0.004	**0.030**	–	–
Uric acid	− 0.47	**0.014**	− 0.195	**0.014**	− 0.403	**0.018**
Ferritin	− 0.01	0.063	− 0.002	0.376	− 0.010	**0.003**
C–reactive protein	− 0.10	0.683	0.035	0.683	− 0.514	**0.017**
Visceral Adipose Tissue	− 0.01	0.105	− 0.007	0.179	− 0.018	**0.003**
Family history of T2D	− 0.07	0.845	− 0.173	**0.025**	0.487	0.159
Family history of SAH	0.49	0.179	− 1.235	**0.008**	0.107	0.759

^a^Correlation coefficient obtained by mixed linear regression model

These associations suggest that multiple metabolic factors contribute to insulin sensitivity, particularly in patients with higher baseline insulin resistance.

**Fig. 3:**
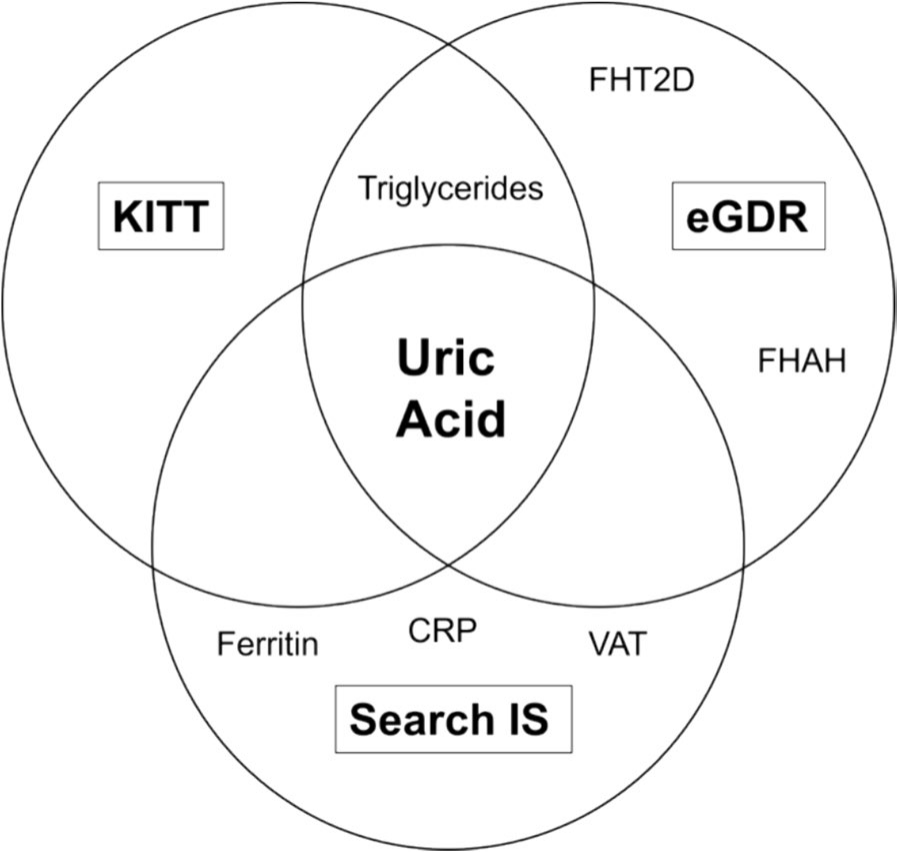
Venn diagram showing the associations between cardiovascular risk factors and insulin sensitivity indices (FHT2D: family history of type 2 diabetes, FHAH: family history of arterial hypertension, VAT-visceral adipose tissue, CRP: C-reactive protein)

In addition to the effect of the variables shown in Table 3, The mixed linear regression model allowed for comparison between the obese and other groups, identifying significant correlations with changes in eGDR during follow-up, including serum total cholesterol (coefficient 0.066, p = 0.006), LDL-C(coefficient 0.066, p = 0.006), HDL-C (0.064, p = 0.008), ferritin (0.071, p = 0.005), CRP(0.065, p = 0.011), insulin/weight dose (0.065, p = 0.011), visceral adipose tissue (0.065, p = 0.011), triglycerides (0.059, p = 0.013), and uric acid(0.076, p = 0.001) (Table 3). Notably, uric acid was the only variable consistently related to all three insulin sensitivity indices (Fig. 3), highlighting its potential role in the pathophysiology of insulin resistance in T1D.

A Venn diagram was constructed to correlate family history, visceral adiposity tissue, and metabolic parameters with the insulin sensitivity indices studied. Therefore, we observed that uric acid was the only common variable related to the three indices evaluated (Fig. 3).

In terms of adverse effects, approximately 25% of the participants reported mild gastrointestinal symptoms associated with metformin use, and no significant differences were observed between the different weight groups (p = 0.893). There were no reports of serious adverse events, and no increase in the incidence of hypoglycemic events was observed during the study period.

## Discussion

The results of our study provide valuable insights into the role of metformin as an adjunctive therapy to insulin in young adults with T1D.Our findings suggest that metformin can significantly improve insulin sensitivity, as evidenced by increases in insulin sensitivity indices such as K_ITT_, eGDR, and SEARCH IS scores. However, this effect was most pronounced in the obese subgroup, highlighting the potential for metformin to play a more significant role in T1D management among patients with higher BMI. Additionally, the study identified uric acid as a potential marker of insulin resistance in this population, providing new perspectives on the multifactorial nature of insulin resistance in T1D.

Our study aimed to characterize metformin as an adjunctive therapy in a population of young adults with T1D with different BMIs but similar ages, insulin doses (U/kg/day), glycemic control and time since diagnosis, in contrast to several studies in this way.

In our study, with the requirement of adequate glycemic control revealed that the obese group showed a high blood pressure, alongside low insulin sensitivity indices, findings that align with secondary analyses from the DCCT.In that study, individuals who gained excessive weight required higher doses of exogenous insulin per day, had elevated blood pressure and non-HDL-C levels [17], and after 13 years of follow-up, they lost previous cardiovascular protection associated with intensive glycemic control and were at a similar risk to people with conventional glycemic control at the beginning of the study [18].

After 26 weeks of adjunct metformin treatment in our study, we observed a reduction in HbA1c of − 0.41% (p < 0.001) in the obese/overweight group but a nonsignificant reduction in the normal weight group. The greatest reduction in HbA1c was specifically in the obese group (− 0.62% [p = 0.004]). This finding was superior to that found in the REMOVAL study, in which approximately 77% of the patients with T1D were overweight and obese, a reduction in HbA1c of − 0.13% was detected in 3 years, and the greatest reduction (− 0.24%; p ≤ 0.001) occurred within the first 3 months of treatment [[12]. However, other studies in adolescents with T1D showed different results; for example, the effect was not sustained at 6 months of follow-up [13], or there was no effect [19].

Therefore, the improvement in weight reduction is also consistent with the effect found in the REMOVAL study [11], in which we found a reduction of 3.01 kg in the obese T1D group. Regarding the lipid profile, our research did not reveal the same benefit of REMOVAL. However, it is important to note that when comparing a similar period, 26 weeks, REMOVAL had little effect on LDL-C levels. Therefore, the difference may be due to the longer duration of statin use (82% vs. 22%) in these two studies. However, a randomized clinical trial in overweight/obese adolescents with T1D revealed a direct influence of metformin on adiposity (− 2 kg) and insulin dose and no effect on cholesterol levels [12].

The variability in response to metformin observed in our study is consistent with the with the broader literature, which emphasizes the need for individualized therapeutic strategies in T1D, especially in those with overlapping characteristics of T1D and T2DM, often referred to as "Double Diabetes." Our findings indicate that metformin is particularly effective in improving insulin sensitivity and metabolic parameters in obese T1D patients, suggesting that even in the context of insulin deficiency, insulin sensitizers like metformin can be beneficial. This is supported by our observation that metformin not only improved glycemic control but also contributed to weight reduction in the obese group, which is consistent with previous studies.

Interestingly, despite the common association between high BMI and increased insulin resistance, we observed that individuals with a BMI over 30 were receiving less insulin per kilogram of body weight compared to those with normal weight. This paradox could be explained by compensatory mechanisms that enhance peripheral insulin sensitivity in obese individuals with T1D, such as higher levels of adiponectin [20] or lifestyle factors like increased physical activity and dietary adjustments. Furthermore, the type of fat gained with insulin therapy in T1D and the profile of interleukins involved in the autoimmune process may differ from classical T1D, contributing to variations in insulin requirements. These observations suggest that the relationship between BMI and insulin resistance in T1D is complex and warrants further investigation [21, 22].

Furthermore, we observed an increase in insulin sensitivity in K_ITT_ in overweight/obese individuals after metformin treatment. This finding is particularly significant as a study conducted in adolescents with T1D (40% with BMI in the 90th percentile) reported improvements in insulin sensitivity, as well as aortic and carotid health, along with reductions in body weight, fat mass, and insulin dose after three months of metformin use [23].

Compared to the other insulin sensitivity indices studied, we found that overweight/obese participants had a low baseline eGDR (6.79), and after using metformin for 26 weeks, the improvement (7.52) was significant. However, this improvement was not enough, as a previously published study highlighted that during a 7.1-year follow-up period, individuals with an eGDR < 8 showed an increased risk of death compared to those with an index ≥ 8 [24]. According to the Pittsburgh Epidemiology of Diabetes Complications Study, a low eGDR (and therefore high insulin resistance) is associated with an increased risk of nephropathy [25], peripheral vascular disease, and coronary artery disease [26].

We also investigated which variables could influence the eGDR in the obese group after metformin use and found that triglycerides and uric acid might be determining factors. The association of triglycerides and uric acid (even within the normal range), with the indices that assess insulin sensitivity, highlights the serum parameters that can be evaluated in individuals with T1D to optimize treatment beyond glycemic targets. In the DCCT and Pittsburgh (EDC) studies, triglycerides were associated with increased cardiovascular risk [27, 28]. Additionally, a doubling in plasma uric acid levels in individuals with T1D during a follow-up of 5.2 years was associated with a risk of a decrease in eGFR (estimated glomerular filtration rate) of ≥ 30%, increased chances of cardiovascular events, and mortality [29]. Uric acid amplifies the effects of elevated glucose levels that stimulate triglyceride accumulation in hepatocytes and contribute to insulin resistance [30]. These findings suggest that these markers could be useful in identifying patients who may benefit most from metformin therapy, thereby optimizing treatment beyond just glycemic control. The association of these markers with insulin sensitivity indices like eGDR further supports their potential role in guiding therapy in T1D.

Metformin was generally well-tolerated by most participants, with only mild gastrointestinal symptoms reported, which improved after a temporary dose reduction, and no increase in the incidence of hypoglycemic events was observed. Additionally, no serious adverse events occurred. While our study has several strengths, including its focus on a specific T1D phenotype and the use of multiple insulin sensitivity indices, it is important to acknowledge its limitations, such as the small sample size and the short duration of the study. These factors may limit the generalizability our findings. Nonetheless, our study provides important insights that could inform future research, particularly in the design and methodology of larger, long-term randomized controlled trials.

## Conclusions

In conclusion, this study provides valuable insights into the effects of metformin as an "insulin-sparing" agent in the treatment of T1D, particularly in obese individuals. The response to metformin varies depending on the patient's weight and insulin sensitivity, highlighting the need for a personalized approach to T1D management. Further research is necessary to explore the heterogeneity of insulin resistance in T1D and to determine the most effective treatment strategies for different patient subgroups.

## Data Availability

The datasets generated and was analysed during the current study are available in the UNIFESP repository. This study was part of Luana A. L. Ramaldes' master's thesis, datasets generated and was analysed during the current study are available in the institutional repository of the university (Unifesp) at the following link: https://repositorio.unifesp.br/items/d05884bc-9ea6-4217-b5fa-c7999c5f2fdf.
